# CUBES: A practical toolkit to measure enablers and barriers to behavior for effective intervention design

**DOI:** 10.12688/gatesopenres.12923.2

**Published:** 2020-01-06

**Authors:** Elisabeth Engl, Sema K. Sgaier

**Affiliations:** 1 Surgo Foundation, Washington, District of Columbia, 20001, USA; 2 Department of Global Health & Population, Harvard T.H. Chan School of Public Health, Boston, MA, USA; 3 Department of Global Health, University of Washington, Seattle, Washington, USA

**Keywords:** Intervention design, implementation science, behavior change, behavioral drivers, behavioral models, research methods, global health, global development.

## Abstract

A pressing goal in global development and other sectors is often to understand what drives people’s behaviors, and how to influence them. Yet designing behavior change interventions is often an unsystematic process, hobbled by insufficient understanding of contextual and perceptual behavioral drivers and a narrow focus on limited research methods to assess them. We propose a toolkit (CUBES) of two solutions to help programs arrive at more effective interventions. First, we introduce a novel framework of behavior, which is a practical tool for programs to structure potential drivers and match corresponding interventions. This evidence-based framework was developed through extensive cross-sectoral literature research and refined through application in large-scale global development programs. Second, we propose a set of descriptive, experimental, and simulation approaches that can enhance and expand the methods commonly used in global development. Since not all methods are equally suited to capture the different types of drivers of behavior, we present a decision aid for method selection. We recommend that existing commonly used methods, such as observations and surveys, use CUBES as a scaffold and incorporate validated measures of specific types of drivers in order to comprehensively test all the potential components of a target behavior. We also recommend under-used methods from sectors such as market research, experimental psychology, and decision science, which programs can use to extend their toolkit and test the importance and impact of key enablers and barriers. The CUBES toolkit enables programs across sectors to streamline the process of conceptualizing, designing, and optimizing interventions, and ultimately to change behaviors and achieve targeted outcomes.

## Introduction

Interventions that aim to shift what people do, and the choices they make, are a major focus of global programs and policy. With aggressive targets set by the United Nation’s Sustainable Development Goals and limited funding for global development, programs must leverage limited resources effectively and efficiently ^i^. To achieve global development outcomes, such as reducing maternal mortality or the number of HIV infections, a plethora of diverse interventions have been designed and implemented across the world that ultimately all aim to shift behavior. Examples include increasing the demand for polio vaccinations in India through mobilization activities ^1^, developing checklists for nurses in Uttar Pradesh, India, to increase adherence to labor and delivery guidelines ^2^, and creating incentives to drive voluntary male circumcision for HIV prevention in Kenya ^3^.

Not all these examples have resulted in successful and sustainable behavior change at the levels needed to have the desired impact on development outcomes. Creating lasting change through successful interventions is hard. First, it requires a thorough understanding of *why* a target behavior is currently not occurring in a given context. Designing and evaluating interventions in the field without a thorough understanding of the underlying drivers of the target behavior in question can be an inefficient use of time and resources ^4^. Second, since people’s choices and behaviors are influenced by many factors, a single intervention is often insufficient to drive change. For instance, a failure of malaria net uptake may be rooted in a lack of availability or accessibility. But this external context is just one part of the picture: beliefs on the part of the end-user, for example about the benefits and risks of the nets, along with many other factors, may also influence decision-making. All these factors must be addressed through a well thought-through set of complementary interventions (an intervention portfolio) to significantly improve the usage of bed nets. Third, not all people are the same. Sub-groups of people within the target population can be differentiated by the varying drivers behind their behavior, necessitating a different set of interventions to be targeted at each subgroup ^5, 6^.

The right levers to focus on, and the portfolio of targeted interventions to scale, are often far from obvious. Programs need a holistic and practical behavioral framework that accounts for and structures all types of barriers and enablers of behavior. Many models of behavioral enablers and barriers exist, but most focus either on systemic drivers, without addressing how individuals can be motivated to respond to such changes ^7– 9^, or on people’s beliefs, personality characteristics or cognitive biases, neglecting context ^10– 19^. Several approaches, such as the COM-B behavior change wheel, the Fogg model, and MINDSPACE, focus most strongly on the appropriate types of interventions to change behavior ^20– 22^. In global development, several organizations such as PSI, Johns Hopkins’ Center for Communication Programs, and FHI360 have created behavioral models incorporating various subsets of drivers. Being application-focused, they usually place great focus on incorporating guidelines on implementation design and monitoring, communication, and advocacy, or on providing a rich compendium of intervention options ^ii^. However, no current behavior change framework helps programs select the right research tool to assess an intervention’s components. Programs need a repertoire of validated methods that will help them assess distinct enablers and barriers of behavior change in the field, because not all methods are effective at measuring each type of enabler and barrier, and only a limited set of methods are used in the development sector today.

In this paper, we provide programs with a practical two-part toolkit to help programs design an effective portfolio of interventions. We call the toolkit CUBES: to Change behavior, Understand Barriers, Enablers, and Stages of change. First, we present an evidence-based framework for understanding behavior. The framework synthesizes stages of change, contextual and perceptual drivers (which can act as enablers or barriers), and layers of influencers, using evidence from multiple sectors. The components were first articulated and applied in the voluntary medical male circumcision program ^23^, and we later refined the framework through a thorough evaluation of existing behavioral models, and by testing its applicability and practicality in several large-scale development programs. Second, to help programs generate actionable insights into the components of the framework in their own context, we recommend a set of research methods from various sectors and detail their strengths and weaknesses, expanding the methodological toolkit of qualitative interviews and quantitative surveys that many programs use by default. We support this with a decision tree to aid the choice of research method according to the practitioners’ specific development program and context.

Real-world programs aim to drive change in complex dynamic systems of people, places, and information channels, and CUBES can be applied at any level in the system. Ultimately, we encourage practitioners use the toolkit to:

1) Understand determinants of behavior: barriers and enablers, both perceptual and contextual.
1) Design idea-channel interventions that address barriers and leverage enablers.
3) Design to all levels of change – individual, family, society, and systems.

To illustrate the usability of the proposed approaches, we present a case study showing how the CUBES framework and the methods toolkit were applied in a large-scale program for voluntary medical male circumcision in Africa. While we have developed this approach through the lens of our programs in global development, its principles can be applied to any behavior change context.

## Methods

### Constructing a best-practice framework of behavior

Grounding intervention design in a comprehensive and actionable behavioral framework is important. Many such models exist, with varying levels of evidence supporting their components. We surveyed models that were a) most influential and b) had an evidence base confirming that they predict behavior, rather than a comprehensive survey of all models in existence. We defined ‘influential’ as having been used to guide behavioral-intervention design across sectors, including health behaviors, and ‘evidence-based’ as the existence of original research evaluating the predictive power of individual components of a model (such as ‘perceived severity of risk’ ^24^ or ‘conscientiousness’ ^12^) on behavior.

We began with a list of models known to the authors, then surveyed key approaches cited in these models, then searched PubMed and Google Scholar for the following terms: ‘behavior’ AND (model OR framework OR drivers OR barriers OR facilitators OR enablers), and focused on the first five pages of results in the search engines. The main search was performed between October and December 2016, with additional targeted searches until December 2018. We identified 17 models fitting the criteria, each focusing on a different set of behavioral drivers ( Table 1). The drivers for which we found experimental evidence of moderate to high predictiveness on behavior were then placed into a framework. We subjected the drivers included in the framework to critical review by four experts in behavioral science, health psychology, and the development sector, focusing on the drivers’ comprehensiveness and applicability to global development. Finally, we applied the framework to design research in our own large-scale programs (see sample case study in this paper) to test for actionability and to further refine its components.

**Table 1.** Behavioral models surveyed, and their main advantages and limitations.

Models of behavior surveyed			
	Origin sector	Main advantage	Main limitation
**Main focus: perceptual drivers**			
**Health Belief Model**	Psychology, public health	Very widely used, wealth of data demonstrating that components explain some variance of behavior.	Neglects factors other than beliefs (biases, emotions, habits) and context/environment.
**MINDSPACE Checklist**	Public policy (interdisciplinary influences)	Concrete, practical checklist of evidence-based techniques to effect change across many sectors.	Focuses almost entirely on unconscious processes and corresponding nudges.
**Integrative Model of Behavioral** **Prediction/Reasoned Action** **Approach/Theory of Reasoned** **Action**	Psychology	Differentiates between different kinds of beliefs.	Context/environment is only accounted for superficially. Does not elaborate on how beliefs are formed; neglects intention–action gap (focus on intention, but intentions do not equal actions) and unconscious processes (e.g. biases).
**Transtheoretical Model** **(Stages of Change)**	Psychology	Change-as-process over time is unique component.	Evidence for six clearly delineated stages of change is weak.
**Health Action Approach**	Psychology	Stages of change extended to repeat behaviors.	No recognition of biases or contextual factors.
**Self-determination Theory**	Psychology	Differentiates between extrinsic and intrinsic motivations and names drivers for intrinsic motivation.	Focused on only one aspect of decision-making: ignores all non-motivational individual and systemic factors.
**OCEAN model of Personality**	Psychology	Trait-based models of personality reliably explain part of the variance in (health) behaviors.	Factors only account for part of an individual’s personality, which in turn only accounts for parts of their behavior. Personality has limited predictive power for a specific behavior, but rather for patterns of behavior.
**Theoretical Domains Framework**	Psychology	Validated and extensive list of barriers and facilitators.	Biases and personality mostly absent.
**COM-B ('capability',** **'opportunity', 'motivation' and** **'behavior')**	Psychology	Emerging from the Theoretical Domains Framework, the first model to link different intervention and policy categories to behavioral drivers in a systematic and parsimonious way.	Limited dimensions of drivers of behavior makes the model easy to understand, but it does not provide much detail.
**Fogg Behavior Model**	Psychology	Similar to COM-B: behavior is understood as a mixture of motivation, ability, and prompts. Uniquely, strong focus on characteristics of contextual cues that are most effective in shifting behaviors.	Model’s view of motivation and ability is simplistic.
**Expected Utility Theory and** **Prospect Theory**	Behavioral economics	Gives insight into appraisal process of a decision.	Accounts for a small subset of drivers of behavior.
**Collection of cognitive biases** **and heuristics**	Behavioral economics, psychology, neuroscience	Insight into ‘automatic’ and unconscious drivers of behavior.	Accounts for only one aspect of decision-making.
**Evo-Eco Approach**	Evolutionary biology, neuroscience	Evolutionary aspects of behavior and embodiment given due importance (e.g. disgust as a primal emotional reaction).	Views behavior as largely caused by automatic/habitual processes.
**Main focus: contextual drivers**			
**Social-Ecological Model**	Psychology	Shows the dynamic ways that different strata of the social sphere influence each other.	Does not account for perceptual drivers of behavior.
**Social Cognitive Theory**	Psychology	Shows how social influence can mediate some perceptual drivers.	Focuses most on self-efficacy, little emphasis on context.
**Practice Theory**	Sociology, anthropology	Focuses on environmental constraints on behavior.	Neglects individuals, focus on theoretical level rather than testing components’ explanatory value.
**Diffusion of Innovations Theory**	Communication studies/ sociology	Clear guidance on techniques to reach different segments of a population to adopt a novel behavior.	Segments individuals in a specific way (how receptive they are to an innovation), does not account for other environmental and cognitive factors driving decision-making.

### Assessing and developing a curated set of research methods to measure drivers

To help programs select appropriate research tools to capture enablers and barriers to behavior, we surveyed methods used across disciplines, using literature research and expert conversations. Unless otherwise mentioned below, we then applied and tested these methods in various combinations in our own large-scale development programs to assess each method’s feasibility, strengths and weaknesses in insight generation. In Zambia and Zimbabwe, we investigated voluntary medical male circumcision ^4, 5, 23^, and in different areas of India we conducted programs on household behaviors relating to maternal and child health (family planning, antenatal care, institutional delivery, postnatal care), tuberculosis care-seeking behaviors, and healthcare provider and front-line worker behaviors within medical facilities and communities (unpublished reports).

## Results

### Cubes: a practical framework of behavior

**Figure 1.**
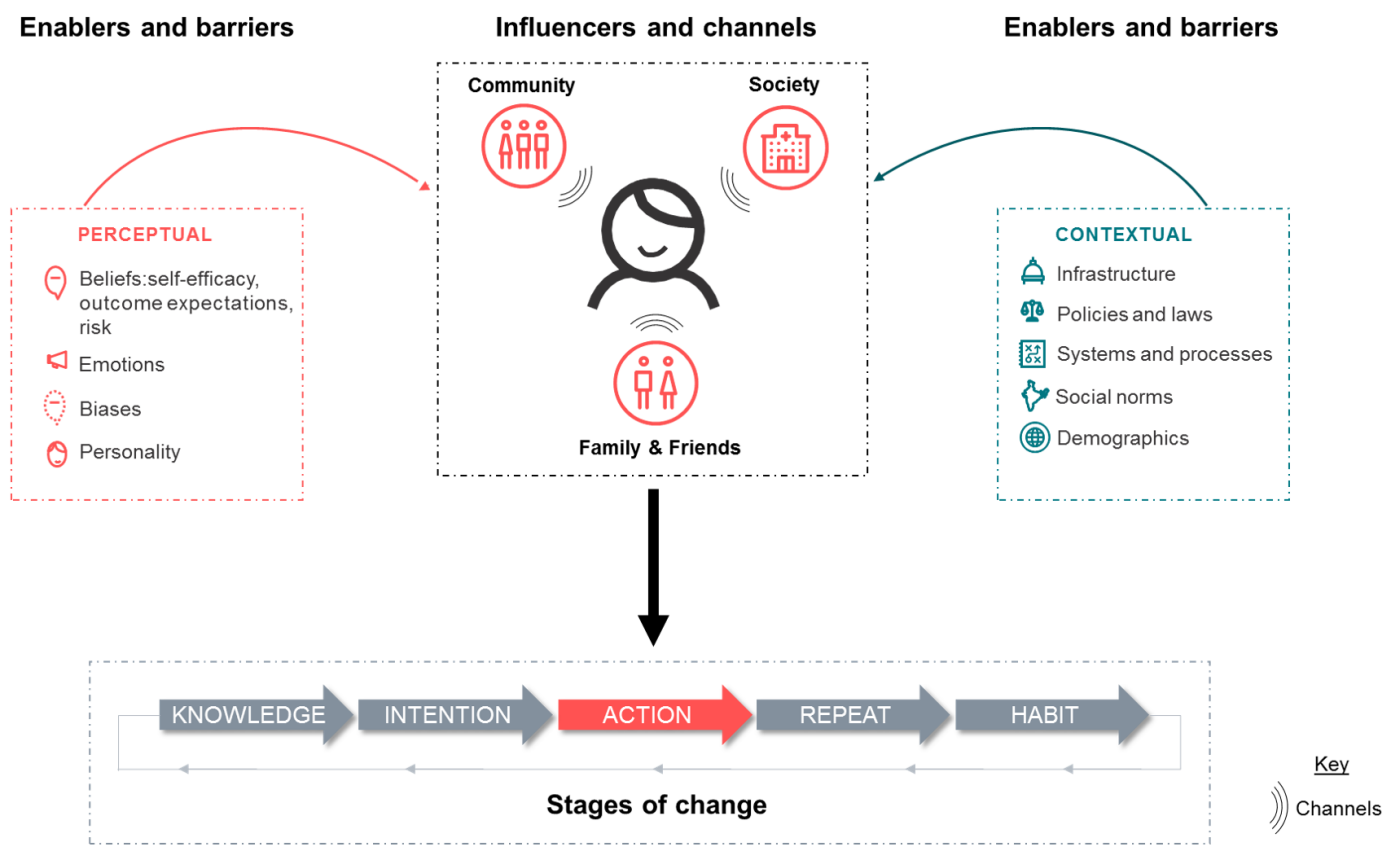
The CUBES behavioral framework. Contextual and perceptual drivers combine to act as enablers and barriers along an individual‘s path from knowledge – encompassing awareness and skills – to intention (or motivation to act towards a goal) and action and beyond. Layers of influencers can affect these drivers and reach an individual through various channels.

Following our review of influential models of behavior, we distilled their most evidence-based components into a practical behavioral framework that programs can use to evaluate existing evidence, conduct research to close evidence gaps, and ultimately design interventions to match barriers to behavior ( Figure 1). The CUBES framework articulates three critical components of behavior change. First, the path toward a target behavior consists of a series of distinct stages. Second, the progression through each of these stages is influenced by a set of contextual and perceptual drivers. Third, these barriers and enablers may be transmitted to the individual, reinforced, or weakened through influencers (such as friends, family, or community members), either directly or through media channels. Below we outline each component and the contributions of the behavioral models surveyed ( Figure 1).

### Stages of the behavioral change process

Both contextual and perceptual drivers influence whether individuals possess the knowledge needed for behavior change, intend to act, or are already acting. These drivers either hinder or facilitate progression along the stages of change.

In global development, many intervention programs focus on enhancing awareness (passive knowledge) and skills (active knowledge). However, it is possible to be aware of an option, and even have the skills to do something, without intending to take advantage of it. For example, in Uttar Pradesh, India, increasing nurses’ skills did not always increase their practices accordingly ^25^. Clearly, knowledge does not equal action. Therefore, understanding where people are on the pathway to behavior, and why they are not moving forward, is key to designing interventions that can move people toward action. This is an essential first step for program designers to orient themselves when evaluating behavior.

The Transtheoretical Model ^13^ proposed a series of relatively rigid stages: precontemplation (being unaware), contemplation, preparation, action, maintenance, and termination, and adds a set of interventions (‘processes of change’) to help an individual progress from one stage to the next. However, the efficacy of assigning individuals to very detailed stages has been called into question in several systematic reviews ^26– 28^. In line with the Health Action Process Approach ^16^, the CUBES framework therefore divides the behavioral change process more simply into three stages of knowledge (ending with the necessary awareness or skills to engage in a behavior), intention (which can also be understood as a plan of action towards a specific goal ^29^), and action. While the path to action and beyond can be understood as a sequence, people can also move back and forth between already-reached stages.

Almost no models of behavior focus on repeat behaviors and habits, despite the importance of sustained change in real-life contexts. Different drivers become more or less important for repetition and habit. The intention to repeat a behavior becomes more likely when two factors converge: a positive evaluation of the previous experience (‘experienced utility’), and a revised self-efficacy in comparison to what was expected ^16, 30^. However, merely repeating a behavior does not create habits. The creation of a habit requires the development of automaticity (the behavior is performed with low awareness, control, attention, or intention) and an association of a behavioral response with contextual cues and an experience of reward ^31– 33^. Some behavioral drivers that can be targeted for single actions, such as intentions, goals, or beliefs, are much less important to the formation of a habit ^16, 31, 33^. For example, delivering a child in a health facility is a one-time behavior, for which intention, goals, and beliefs matter greatly, but exclusive breastfeeding after the child is born is close to a habit, which after initiation does not require a woman to form the intention from scratch every single time. Instead of targeting beliefs and intentions, then, restructuring of environmental cues and conscious inhibition of unwanted habits may have greater success in creating lasting habits ^34, 35^.

### Contextual drivers of behavior

Behavior emerges out of a complex system of interactions between individuals and the systems they act in. The Social-Ecological Model introduces the concept of ecosystems, which examines the dynamic ways that different layers of the social sphere influence each other ^8, 36, 37^. Adapting the Social-Ecological Model ^8, 36, 37^, social norms and customs influence individuals in an ecosystem in several layers. These norms may be explicit, but they can also emerge from an implicit layer of what Practice Theory defines as ‘shared cultural knowledge’ that expresses itself as routines and habits ^7, 38^. Social norms are a construct that can only exist on the level outside the individual: through collective behavior and ‘shared knowledge’, norms describe a set of practices of what other people do (descriptive norms), or prescribe what people should do (prescriptive norms). Both of these may influence attitudes and behavior ^39^. Unlike individual-level beliefs, norms usually imply some consequence to the individual should they deviate from the norm, such as disapproval ^40^.

Structural factors are further contextual drivers that may shape and constrain perception and action. These aspects are usually not fleshed out in behavioral models. However, unless these constraints are removed, interventions acting on perceptual drivers will not allow for the target behavior to occur. We propose differentiating between infrastructure, policies and laws, and systems and processes, all of which vary strongly with their respective context. For example, infrastructure drivers may include availability and condition of roads leading to a health facility, equipment to perform a test, or seeds for farmers to use. Policies and laws constraining behavior can exist on several levels, from national laws to facility-level guidelines. Systems and processes could mean supervision, training, feedback, or incentive systems under which healthcare providers operate in a facility, or the tools teachers have available to plan lessons and receive feedback on their tuition. Finally, demographic factors such as age or education level act as contextual constraints on behavior.

### Perceptual drivers of behavior

Multiple models recognize that behavior is also shaped by perceptual drivers that, together with contextual drivers, combine to make a target behavior more or less likely.

***Biases and heuristics.*** Most aspects of an individual’s cognition and behavior are influenced by ‘automatic’, often unconscious, mental shortcuts or rules of thumb (heuristics) that can bias decision-making. Many biases can be difficult to change, but knowing about them makes it possible to construct environments where the best decisions are the easiest. A typical example is setting the default option of a pension program to ‘opt-out’ instead of ‘opt-in’: staying in a pension program after auto-enrollment is easier than making the effort to join in the first place ^41, 42^. Examples of heuristics and biases are the optimism, confirmation, and availability biases, anchoring-and-adjustment, hyperbolic discounting heuristics, and the status quo and representative biases ^17, 18, 41– 47^. MINDSPACE, an influential checklist for behavior change developed in part by UK government agencies ^20, 48^, is an example of using biases (such as a bias to choose the default option) as tools to design interventions.

***Beliefs.*** Beliefs are formed by learned experience, differentiating them from biases (which humans share to varying degrees as a result of how our brains evolved). In the Integrative Model of Behavioral Prediction/Reasoned Action Approach, beliefs about what outcome can be expected from a behavior (called ‘attitudes’ in that model), normative beliefs (how others will judge a behavior), and beliefs about the extent of one’s control over the behavior (self-efficacy) all influence intention, which is seen as the main driver of behavior ^19, 49, 50^. Experiments have shown that some beliefs predict behavior better than others. For example, perceived control (self-efficacy) and beliefs about a behavior’s outcome are better predictors of behavior than normative beliefs ^51, 52^. Indeed, self-efficacy beliefs have emerged as a strong predictor of behavior across other models, such as Social Cognitive Theory, the Health Belief Model, and the Transtheoretical Model. The strong influence of self-efficacy on behavior has been shown experimentally in many studies ^53– 57^.

Outcome expectations are another example of a belief. An individual appraises a potential behavior by weighing perceived costs and perceived benefits, which may be emotional (‘How will the outcome of the behavior make me feel?’), social (‘How will others judge me?’), or functional (‘How does this help or hurt me?’). Outcome expectations, together with the perceived severity of an outcome, the perceived susceptibility to that risk, and self-efficacy, are central to one of the most widely-used models of behavior, the Health Belief Model ^14, 24, 58^. Evidence from several systematic reviews shows that increasing perceived benefits and decreasing perceived costs to a behavior will be most likely to cause an individual to engage in the target behavior ^10, 24, 59, 60^. Beliefs around (professional or social) self-identity may also be predictive of behavior. For example, environmental self-identity, or seeing oneself as ‘a person who acts environmentally-friendly’, is related to several environmental behaviors ^61^. However, empirical research on identity and behavior is still emerging.

***Emotion.*** The experience of emotion (affect) also drives behavior. Affect arguably colors all perception and powerfully shapes decision-making ^20^. While affect is used as a distinct driver of behavior in the MINDSPACE checklist ^20^, the COM-B framework ^22^ includes it as a rapid, automatic component of motivation. One example of targeting affect to drive motivation to engage in a new behavior can be seen in an intervention promoting soap use in Ghana: education around the benefits of soap did little to drive up its popularity, but emphasizing the feeling of disgust from ‘dirty hands’ resulted in significantly increased soap use ^62^. This emphasis on automatic emotional responses, such as disgust or a desire to conform with others, is a key component of the Evo-Eco behavioral model ^63^.

***Personality.*** Personality traits are not often included in models of behavior—not even in the Theoretical Domains Framework, which includes the perhaps most comprehensive list of barriers and facilitators ^64^ —but they can strongly influence an individual’s propensity to engage in and maintain health behaviors ^65, 66^. Current applications of personality models to behavior prediction focus on aggregates of behavior, or behavioral patterns of behavior such as going to check-ups and regular physical activity (preventive health behaviors). Such models often do not attempt to predict single instances of behavior, which has been shown to be much less reliable ^65^. Currently, the dominant personality model with a large evidence base behind it is the so-called Five-Factor Model ^12, 66– 68^ with the five broad traits of openness to experience, conscientiousness, extraversion, agreeableness, and neuroticism (‘OCEAN’). Conscientiousness appears to be an especially strong predictor of behavior patterns, such as sticking to preventive health routines ^65, 66^.

### Influencers and channels

Following from the Social-Ecological Model ^8, 36, 37^, influencers surround an individual in layers. Family and friends or peer groups are the closest layer to the individual. The next layer usually consists of relationships in the community, for example in workplaces, schools, and neighborhoods. The most distant layer is the larger social context. Influencers can reach individuals either directly or at scale via various media channels, which is important information to determine how to deliver interventions. For example, female self-help groups can serve as a channel for rural women in India to reinforce or change social norms relating to a certain target behavior. For an intervention to work, the content, the type of influencer, and the channel through which they reach individuals must be identified as relevant to the target individuals. Finally, social influence is often not intentional as a self-help group might be, but less explicit influence may not be any less powerful.

### Interaction between the ‘building blocks’ of CUBES

Depending on the behavior, all the drivers mentioned above will be relevant to varying degrees to any one individual, and their combination will result in a larger or smaller tendency to act. The elements of CUBES influence each other deeply. For instance, punitive supervision (systems and processes) by medical-officers-in-charge (influencers) in a health facility might lead nurses on the receiving end to experience high anxiety (emotions), and to beliefs that trying hard will not result in any benefit to them (outcome expectation belief). It is important for programs to disentangle these components, even when they influence each other, because this determines what kind of intervention is best placed at what level.

Therefore, the enablers and barriers in the different ‘building blocks’ of CUBES can be seen as a checklist for programs that they can utilize to design effective interventions.

Models of behavior that simplify to the point of ‘motivation’ or ‘ability’ (such as COM-B or the Fogg model) are simpler, but also less actionable for programs on the ground. For example, if a study finds a lack of motivation to go to the doctor despite having symptoms indicative of tuberculosis, this alone is not actionable by an intervention. Instead, programs need to know where potential patients are on the knowledge–intention spectrum (they have clearly not yet taken action), whether they perceive their symptoms and the disease as a danger to their and others’ health (risk perceptions), whether they think going for a check-up will actually alleviate symptoms (outcome expectations), whether they feel able to skip work and other responsibilities to attend an appointment (self-efficacy), whether appropriate facilities are even available, affordable, and accessible (structural factors), and whether there is stigma involved in seeking care (social norms). All these components would feed into the concepts of ‘motivation’ or ‘ability’, but require very different interventions to effect change.

Once CUBES has been used to understand and categorize existing evidence, evidence gaps can be closed in a focused way with primary research. Not all components of CUBES are best captured and intervened on in the same way. In the following sections, we introduce a method mix designed to identify a comprehensive set of drivers.

## Methods of measuring drivers

Qualitative interviews, focus groups, and quantitative surveys are some of the most common methods of insight generation in the global development. These strategies complement each other: qualitative methods are best suited to exploration and capturing nuances, whereas quantitative methods are indispensable for discovering patterns and weighing the relative importance of different drivers, which is essential for developing interventions that address the barriers that matter. Here, we propose two overarching considerations for programs to add value to the methods used.

First, existing methods can be improved by using the CUBES framework as a checklist against survey or discussion-guide items, to check whether a comprehensive set of enablers, barriers, influencers, and stages is captured. Too often, methods such as quantitative surveys remain at the level of measuring practices (or ‘ *what*’ data) and demographics, at the expense of the ‘ *why*’, or perceptual and contextual drivers of the target behaviors.

Second, programs could benefit from expanding their own toolkit of methods by selecting approaches to investigating behavior from a variety of sectors. The right method will depend on what type of data needs to be captured, and whether the purpose of research is exploration or testing specific hypotheses. A method mix can also help counteract any weaknesses of individual methods.

### Choosing the right type of method for different stages of research

Research methods can be divided into descriptive, experimental, and simulated approaches; the last two are relatively under-used in global development. Whether qualitative or quantitative, *descriptive* methods such as interviews or observation aim to describe and explain behavior without testing the effects of manipulating variables systematically. While they can explore ‘how’ and ‘why’ questions (and are therefore often called ‘exploratory’), they do not have the ability to systematically relate the effect of change to outcomes (‘confirmatory’). *Experimental* approaches can be used to test hypotheses and find causal relationships by systematically varying variables and testing their effects on outcomes. However, they lack the ability to survey a broad spectrum of factors at once that exploratory methods provide. Finally, *simulated* methods can assess cause–effect relationships in a virtual environment when experimental field methods are not possible or are too complex, but they rely on many assumptions to construct the simulation.

### Expanding the descriptive toolkit

***Journey mapping.*** Developed and primarily used in market research ^69, 70^, journey mapping systematically tracks people’s experiences and interactions with a product, service, or life event over time, as people form beliefs about the product or event and make decisions, perhaps via influencers, to interact with or avoid it. This method is especially well-suited to get a sense of stages of change. Journey mapping can also help form hypotheses about segments of customers who share distinct characteristics in order to target them with bespoke messages via different channels. In addition, it can be useful in generating hypotheses about underlying behavioral drivers that can then be tested further.

Journey mapping uses many different techniques to collect data, including one-on-one qualitative interviews, focus groups, ethnography, web analytics, customer reports via apps, and (qualitative) network mapping ^69, 71^. Below, we show in a case study how journey mapping was successfully integrated in a program understanding decisions around voluntary medical male circumcision ^5^.

***Observation.*** Observation is a versatile and routinely used tool in global development to collect data about what people do, the context that surrounds them, and how they interact with processes, objects, or each other ^72^. The spectrum of observation techniques ranges from researchers interacting closely with communities ( *participant observation*), to covert or overt ‘ *natural observation*’ without participation, to *controlled observation*, where procedures are highly standardized. Measuring time and frequency of practices of medical residents in hospitals ^73^, or of nurse practices in hospitals in India ^25^, are typical examples of controlled observation. Such *time-and-motion* studies observe the time taken and actions of participants executing distinct components of a process. Observation is also a tool to measure contextual drivers such as infrastructure or processes. For example, *facility and infrastructure audits* commonly combine observations with interviews of key stakeholders to track characteristics such as hospital staff coverage, equipment availability, or communication tools ^74^.

To get a holistic sense of contextual drivers, observation can track more than behaviors or supplies. Instead, immersive observations in the participants’ natural environment could be structured to assess the set of contextual enablers and barriers outlined in the CUBES framework. We call this approach ‘ *structured immersive observation*’. For example, in a recent observational study on nurses in healthcare facilities, we measured key contextual dimensions as follows. First, we assessed facility infrastructure available to nurses: whether equipment and drugs required for routine tests were available at the time of testing, staff coverage throughout the period of observation, availability of beds, water, and electricity, and transport options for patients to be referred. Second, we assessed systems and practices, such as interactions with and feedback from other staff, time spent with patients and tests performed, documentation systems and job aids, and training records. Third, we assessed community norms by observing community interactions and communications with the nurse.

***Enhanced quantitative surveys.*** Quantitative surveys are a critical tool to obtain insights on many enablers and barriers to behavior simultaneously and at scale, and consequently are a mainstay of global development research. Survey design is a broad field with a large array of approaches. Here, we recommend three key techniques that can enhance the design of quantitative surveys to measure potential behavioral drivers. Survey questions can be structured to account for as many components of CUBES as possible. In sensitive contexts, surveys can also be enhanced to counteract respondent biases. Finally, while not within the scope of this article, programs would benefit from leveraging quantitative data for insight beyond descriptive analyses: for example, population segments can be found for targeted intervention design ^5^.

***Driver-structured surveys.*** Programs can use the CUBES framework as a checklist to assess whether a survey captures the range of potential contextual and perceptual enablers and barriers, influencers and channels, and stages of change relating to behaviors of interest. We often find surveys only measure a narrow set of drivers, which presents the opportunity to generate a more holistic view of behaviors of interest and the system. This can also be enhanced by adapting existing validated tools, such as scales testing personality or self-efficacy (see below).

***Standardized scales.*** A simple and high-yield modification to quantitative surveys is the inclusion of previously validated and standardized rating scales relating to CUBES perceptual drivers. Standardized scales that test specific cognitive processes include the Risk Propensity Scale ^75^ and the ten-item General Self-Efficacy Scale ^76^. Types of emotions and their felt strength have also been widely measured with graphical rating scales, such as the Self-Assessment Manikin ^77^. All these scales can be flexibly adapted to specific contexts. For example, in a study investigating women’s propensity to engage in breast cancer prevention, self-efficacy was asked in two items: ‘the extent to which participants were confident that they could conduct breast self-exams every month; and when they conducted a breast self-exam, how confident they were in their ability to identify a “lump that needs medical attention.”’ ^78^.

Personality tests, widely used in the private sector, also use standardized rating scales. Many studies show some predictive value of the OCEAN model’s ‘Big Five’ personality traits on health behaviors ^65^, especially conscientiousness ^66^. Questionnaire designers can tap a large number of validated instruments to test OCEAN components, such as the public-domain International Personality Item Pool ^79^.

All standardized scales have the advantage of using previously validated instruments, and that individual differences can be captured with relatively little effort. A limitation is that, like all self-reports, such tests are susceptible to reporting bias, since participants can deduce or guess socially desired responses (those that the respondent thinks will make them appear in a favorable light). Responses may therefore be compatible with the participants’ sense of self rather than their actual behavior.

***Informal confidential voting interviews and polling-booth surveys.*** Methods that stress anonymity and confidentiality, such as polling-booth surveys (PBS) and the Informal Confidential Voting Interview (ICVI) approach, can be used to probe sensitive topics, as they counteract social desirability bias. The ICVI consists of a one-on-one interview followed by self-completion methods ^80^. Similarly, PBS collects feedback from a group of people who respond anonymously and independently through a ballot box. Comparison of one-on-one interviews with PBS ^81^ and with ICVI ^82^ on sexual risk behavior in Indian men and women demonstrated their value, as more risky behaviors were reported with each method.

***Standardized patients.*** Standardized patients (SPs) are people trained to play the role of a patient with certain medical and personal characteristics, who interact with healthcare providers in a realistic setting. SPs are comparable to ‘mystery shoppers’ in consumer research ^83^, in that the healthcare professional does not know the patient is not real ^84^. In other scenarios, both parties know about the setup ^85^. The SP method has been applied to investigate healthcare provider behavior in many contexts, such as prescription practices of pharmacists ^83^. It has also been used to assess how doctors communicate with their patients, such as how surgeons disclose medical errors to patients ^85^. The SP approach can compare expected with actual behaviors, and analyze communication, such as the content of advice given to patients ^84^. While the method on its own is very well suited to capture practices and contextual drivers such as infrastructure and processes (of patient interaction), other drivers, such as beliefs or biases, are less accessible to investigation.

***Social network analysis.*** Social network analysis (SNA) maps relationships between people, organisms, groups, or devices ^86^. When analyzing behavior, SNA can be an excellent tool to describe which influencers and which channels are most important to transmit certain norms and information. SNA can be both qualitative or quantitative. Data can be generated from surveys, ethnography, or observation, or mined from existing resources such as GPS coordinates or twitter messages ^87, 88^. Many field studies have used SNA to focus on where in the system to intervene. For example, in Uganda, researchers mapped the process of obtaining a diagnosis for tuberculosis through provider and patient networks, and the steps where delays were most common could be identified ^89^. Ultimately, SNA is a flexible and versatile method, but specifically focuses on identifying centers of influence.

***Leveraging ‘passive’ datasets.*** As in the SNA example, insight can also be generated from leveraging ‘passive’ datasets, generated for a different original purpose, without direct interaction with or observation of respondents. Examples are information obtained from GPS, satellites, and sensor systems, as well as other databases. To investigate contextual drivers, satellite images can map physical conditions of the built environment, which can then be related to behaviors and drivers from other datasets ^90^. For some audiences, social media data can be an appropriate source of aggregate estimates of positive or negative sentiment ^91^. More analog data sources can also help generate insight, as in an analysis of Kenyan newspaper articles about voluntary medical male circumcision, which provided insight on the types of risks that were presented to readers ^92^.

### Assessing decision drivers through ‘ *in vitro*’ experiments

We propose that programs can benefit from ‘ *in vitro*’ experimental methods before testing specific interventions in lengthy trials in the field. Experimental methods that track the decisions participants make in laboratory-like conditions serve several purposes: programs can systematically change and test enablers and barriers to behavior, predict behaviors in response to specific interventions, determine those features of a service or product that are most likely to align with the customers, and forecast the market size of a product or features based on predicted behaviors. All these factors narrow down the potential characteristics of an intervention to be tested in the field, and ultimately make the design of effective interventions more probable.

***Discrete choice experiments.*** Discrete choice experiments (DCE), extensively used in market research, uncover preferences and value attribution from the choices that participants make, rather than from the participant disclosing them. They are a powerful tool to predict behaviors ‘ *in vitro*’, assess which features of a product or message are most important to the customer, and to forecast market shares of products. DCE have been shown to be predictive of health behaviors ^93^. For feature selection, participants are typically shown multiple iterations of sets of products with varying features. In each trial, the participant picks one option. For example, a discrete choice experiment in South Africa evaluated which characteristics of HIV prevention products, such as the method of use, or the protection against diseases other than HIV, would be most valued by participants ^94^. From participant choices, a model can be built showing which level and which combinations of a product’s features predominantly drive decisions, where the tipping points of certain preferences lie, and forecasting product market share. DCEs can also be used to test various ‘what-if’ scenarios, and results can then be used as a funnel to select the most promising attributes for a field intervention. Simulated test marketing is a related concept, in which the consumer is asked to make choices in a realistic environment, with similar systematic manipulation of test variables.

***Decision games.*** Purely quantitative DCE approaches are mostly used to cycle through permutations of features and types of products or interventions. However, a related ‘decision game’ method mixing quantitative with qualitative elements can help investigate which behavioral drivers most influence choices made by participants. In a recent study with healthcare providers in Uttar Pradesh, India, we used such a group ‘decision game’: participants were given a set of scenarios, each with a set of response options, and were asked to choose the option they thought other participants would select (unpublished reports). Response options coded for different behavioral drivers, and participants were later qualitatively probed on their choices. For example, nurses were asked which nurse in a scenario was likely to be most stressed: the one working in an understaffed facility (coding for infrastructure-staffing), the one dealing with demands from patient family members (influencers-community), or the one facing constant scrutiny and accountability from supervisors (systems and processes – supervision).

***Implicit attitude tests.*** Few research methods are suitable to measure enablers and barriers that respondents cannot or do not want to report. Over the last two decades, experimental psychology has developed a battery of experimental approaches to measure ‘implicit’ biases, or biases that are inaccessible to conscious awareness and self-report, but nevertheless influence behavior. The underlying concept of implicit attitude tests is that our brains perform unconscious evaluations of concepts, people, and objects, which have arisen from past experiences and cannot be measured by explicit questioning.

The most widely-used implicit attitude test is the Implicit Association Test (IAT). This test is based on the concept that participants can perform a task more quickly when they see two concepts as related than when they do not associate them with each other ^95^. IATs have been used to measure a plethora of social stereotypes, such as gender and racial biases ^iii^, for instance in the Democratic Republic of Congo ^96^. In market research, the IAT has been used to gauge consumer attitudes toward different products ^97^. The predictive value of implicit attitude tests on behavior is still under debate ^98, 99^. For this reason, and because IATs can only test a small set of associations within a test that requires training participants, we only recommend implicit tests when a behavior is likely to be influenced by a specific, deep-seated bias that respondents are unlikely to report.

### Using simulations to model ‘what-if’ scenarios

Simulations have the unique advantage that complex ‘what-if’ scenarios can be explored at the push of a button, which can be used to supplement and inform data collection or to optimize interventions. Through the construction of ‘virtual worlds’, mathematical models can simulate the impact of implementing certain interventions, or of targeting interventions to specific sub-groups. They can also generate hypotheses on what a likely driver for behavior might be. As an example, agent-based models have been used to simulate the large-scale effects that emerge from the actions of many single agents, such as the spread of disease or of social norms and beliefs ^100, 101^. Similarly, Bayesian cognitive mapping builds probabilistic models of the likelihood that agents make certain decisions ^102^. However, simulations are only as good as the model and assumptions that underlie them. The relevant and correct starting parameters must be chosen with caution, which includes a degree of subjectivity ^103^, and generalizations from a model based on specific assumptions are limited. Table 2 summarizes the approaches discussed, and their strengths and weaknesses.

**Table 2.** Overview of enhanced and novel insight generation methods as part of the CUBES toolkit.

Method	Primary insight gain	Most testable CUBES components	Method type	Advantages	Disadvantages
**Descriptive**					
**Journey mapping**	Tracking experiences and influencers over time	Stages of change, beliefs, emotions, influencers and channels	Qualitative	Mapping experience, influencers drivers over time	Self-report
**Observation** ■ Time-and-motion ■ Infrastructure audits ■ ‘Structured immersive observation’ (SIO)	Systematically tracking practices/ duration (time and motion), infrastructure and supplies (audits), and CUBES- structured contextual drivers (SIO)	Contextual drivers: structural, systems and processes; behaviors observed	Quantitative	Behaviors and contextual drivers and barriers can be measured in their natural environment, in a standardized and replicable way	Observed participants and researchers are prone to behavioral or recording biases, respectively.
**Enhanced surveys**					
■ Driver-structured surveys	Using CUBES as checklist aids systematic capture of enablers, barriers, influencers, and stages of change	All	Quantitative	Holistic overview of all potential drivers possible in one dataset per respondent	Not all drivers are equally well captured by self-report
■ Informal confidential voting interview (ICVI), polling-booth surveys	Adding anonymized components encourages responses on sensitive issues	Social norms, beliefs	Quantitative, ICVI also qualitative	Greater disclosure on sensitive issues	Yes/no response format leaves no room to explore; anonymous data can only be analyzed in aggregate
■ Standardized scales	Testing perceptual drivers with validated, standardized tools (e.g. self- efficacy, risk propensity, personality)	Beliefs, personality	Quantitative	Ready-made aids to assessing perceptual drivers and barriers	Prone to self-report bias
**Standardized patients**	Tracking behaviors, context, and interactions through simulated ‘patients’ with a set of standardized characteristics	Contextual drivers: structural, systems and processes; behaviors observed	Quantitative, qualitative components	Standardization allows for comparability, realistic setting and covert data collection for realism	On its own, is mostly limited to ‘what’ data and cannot explore drivers for practices.
**Social network analysis**	Revealing direction and strength of relationships in a system	Influencers, social norms	Qualitative or quantitative	Versatile (qualitative or quantitative), useable for networks of any size and type, unique method of identifying influential targets for potential intervention	Network modeling can only investigate limited drivers and barriers in one network.
**Leveraging ‘passive’** **datasets**	Generating insights from sensor, mobile phone, satellite, GPS, social media, and other databases, with no direct customer interaction	Different, depending on dataset	Quantitative	Large-scale existing datasets can be tapped and integrated with other research methods, ‘bird’s- eye’ view of context possible	Passive nature means no opportunity to probe; existing datasets may not focus on key customer groups
**’ *In vitro*’ experimental**					
**Discrete choice** **experiments**	Participants make repeated choices between a set of options whose attributes are systematically varied, in order to uncover which attributes are most important	All, least useful for biases	Quantitative	Quick to develop, test, and analyze. Participants do not have to explain ‘reasons why’, which are inferred from choices	Correlation of hypothetical with real- world choices is difficult to predict. Providing response options that clearly represent distinct drivers and barriers is not trivial
**Decision games**	Gamified, social experiment version of a discrete choice experiment	All, least useful for biases	Quantitative and/or qualitative	Gamification increases engagement, asking about what other participants select instead of own choices circumvents some respondent biases	Same as discrete choice experiments; qualitative approach is difficult to interpret
**Implicit attitude tests**	Using reaction time in response to tasks and other measurements to determine whether participant sees concepts as related or not	Biases	Quantitative	Unique method to assess strong biases inaccessible to self-report or observation	Method not well tested in low-resource settings, correlation of output and behavior not obvious, each test can only test a limited number of associations
**Simulated**					
**‘What-if’ simulations**	Modeling simulated decision-making or outcomes in response to changing parameters in complex systems	All can be simulated	n/a	Unlimited permutations of changes (‘what-if scenarios’) in a complex system can be modelled	Any model will only be as good as the input data (which does require field-level input), highly specialized skills required

### Choosing the right method at the right time for the right purpose

**Figure 2.**
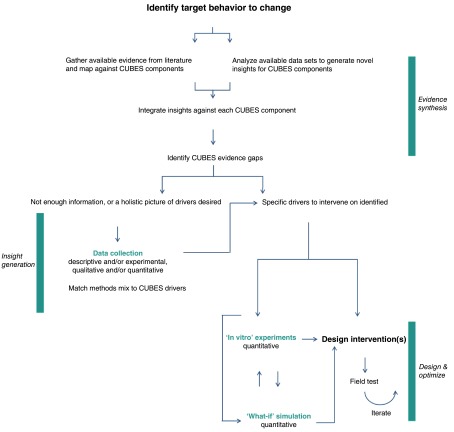
Decision aid for choosing the right research approach at the right time, for the right purpose.

The goal of any program is to implement successful interventions in the field. Figure 2 depicts the process of setting the research agenda, generating insights, and designing and optimizing interventions that programs can use, depending on the knowledge level at the start of the research process. First, when programs define a target behavior to be changed, they can evaluate existing evidence against the components of the CUBES framework. This can be done either from existing literature or analyzed from datasets, or both. To directly choose an intervention that works, programs must already have narrowed down specific drivers to intervene on. This may be the case in a data-rich environment; in other cases, political or resource constraints limit what is testable in the field. In such cases, primary research may not be required or appropriate. On the other end of the spectrum, a program might know what it wants to change—for example, to increase the uptake of modern methods of contraception—but have little systematic knowledge of the types of drivers that may be involved. In this case, exploratory research is warranted (‘insight generation’ in Figure 2). We have found that a quantitative survey often provides the best practical balance between assessing many components of CUBES at scale. Either before a survey (to inform its design) and/or after (to dive deeper into specific findings), specific descriptive or experimental methods offer particular strengths assessing specific CUBES components and can supplement a survey ( Table 2).

Descriptive methods can be qualitative or quantitative, and many can take both forms. To choose a qualitative or quantitative focus, programs can consider whether the freedom to explore limited aspects in depth is most important (which means a greater focus on qualitative research), or representativeness at scale and the relative likely impact of each driver (which points to quantitative methods). Often, programs use preliminary qualitative research to the inform the design of quantitative research, especially in field settings where quantitative research is conducted in person and therefore is expensive and time-consuming, whereas small-sample qualitative research is less resource-intensive. The freer structure of qualitative research also typically allows for follow-up questions and clarification. However, we recommend that this order not be followed by default, but rather examined on a case-by-case basis. In our experience, qualitative research can sometimes divert resources from quantitative research, which can be wasteful if results from a smaller sample cannot be generalized. Instead, qualitative and quantitative research can also be run in parallel, with a complementary focus on different drivers; pockets of qualitative components can be mixed into qualitative research; or qualitative back-checks can be conducted after quantitative research.

In global development, many programs tend to focus on descriptive methods for insight generation, followed by field implementation. In the field, randomized controlled trials tend to be seen as the gold standard for assessing the effectiveness of an intervention, even if they are not always employed in practice. In addition to preliminary evidence synthesis and using a flexible methods toolkit for specific deep-dives, we argue that this approach misses a key step, namely ‘ *in vitro*’ experimental methods. These methods can narrow down the many potential hypotheses emerging from exploratory research, so that only the enablers and barriers likely to be most impactful are ultimately tested in the field. This optimization can be used to choose between different types of interventions (such as monetary incentives versus more information on risks and benefits), as well as the components of a specific interventions (such as the magnitude of incentives likely to be most effective). If rich descriptive data is already available, and so a limited set of specific hypotheses around limited drivers can be formed from the outset, programs can directly skip to this step. This step can also be done in parallel with exploratory research, if evidence is strong on specific drivers but weak on others and a holistic picture is desired. As detailed above, purely simulation-based methods can be of use here to model the effect of many different changes. As a result of this step, field testing will be based on much stronger evidence.

## Applications of the cubes toolkit: case study

### Designing interventions to increase the uptake of voluntary medical male circumcision (VMMC)

**Figure 3.**
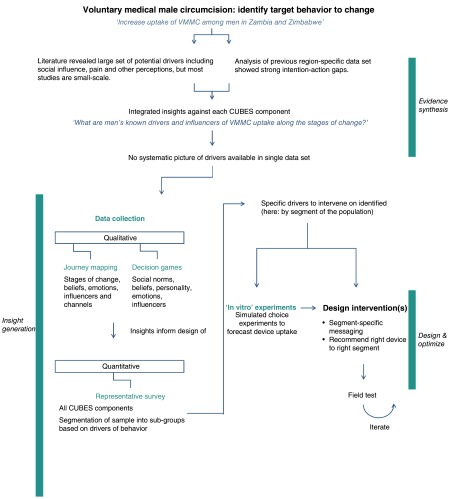
Process of evidence evaluation, insight generation, and intervention design and optimization in a VMMC program.

In Figure 3, we briefly outline the methodological choices made to investigate enablers and barriers to uptake of voluntary medical male circumcision (VMMC) in Zambia and Zimbabwe. VMMC is a highly cost-effective intervention for preventing HIV acquisition that is being scaled up in eastern and southern Africa ^5, 104^. The achievement of the program’s ambitious targets necessitated shifting the behavior of many men in the community who either did not consider circumcision, or if they did, did not take action. Therefore, the program needed to understand the multiple interacting factors that facilitate or inhibit men’s decision to get circumcised, and to test interventions to address those factors. A synthesis of previous studies revealed a variety of existing insights on many behavioral drivers, such as concerns around pain, complications, or cost, as well as patterns of influencers ^105^. Analysis of a large quantitative survey further showed a relatively small awareness-intention gap, but a large drop from intention to action ^106^ A total of 64% of men intended to get circumcised, but only 11% did. However, a holistic view assessing the prevalence of each of these, and other, drivers and their relative strength was lacking, and most research was either small-scale or qualitative ^5^. The existing studies could not answer why there was a strong intention-to-action gap. Also, the research did not examine or reveal the heterogeneity among men—the fact that a given enabler or barrier may be important to one man, while not as relevant to another.

A broad set of CUBES drivers therefore needed to be captured at scale to assess the relative importance of each driver in a single dataset. However, as the stages of change appeared to be of primary importance, journey mapping and qualitative decision games were first used to understand the stages of change and associated beliefs and influencers at each stage in more detail ^5^. In summary, qualitative research pointed to a much more nuanced picture. Men develop positive as well as negative beliefs, influenced by individuals around them, as they move through the various stages of change. These competing beliefs and associated emotions move men towards or away from the decision of getting circumcised in distinct stages of change. For example, beliefs in the early stages include “VMMC protects myself and my partner from STIs” (positive) and “the procedure is painful”. As men move to consider undergoing the procedure, there emerges a strong conflict between the positive beliefs and negative ones, such as circumcision threatening self-identity, leading to distrust between the man and his female partner, and the perceived long healing time. The conflict between emotions such as shame, distrust, and fear and perceived potential benefits move men into a state of cognitive dissonance and stall them from taking-action. However, it was also clear that not all men held all beliefs equally strongly, or were equally subject to the same type and strength of influencers. These insights informed the design of a large-scale quantitative survey investigating CUBES components comprehensively. Having this dataset available then allowed us to segment men on the enablers and barriers to VMMC, so that messaging interventions could be designed targeting the most important drivers for each segment. For instance, among the six segments found in Zimbabwe, Embarrassed Rejecters had mostly negative beliefs about VMMC, as well as fears and concerns regarding the procedure, and had little social support ^5^. However, they did not lack the knowledge that strongly characterized another segment, Neophytes. Accordingly, segment-specific interventions, including messaging through front-line workers or media campaigns, could be developed. In addition, the roll-out of circumcision devices as an intervention to improve the uptake of circumcision was an important consideration for programs. However, it was unclear what the potential demand and market share of these devices would be. The demand for different devices to carry out the VMMC procedure was forecast using simulated test marketing, a technique related to discrete choice experiments, so that the right devices could be marketed with the right message to the right people ^104^. Interventions designed based on this research are currently being piloted at national scale in Zambia and Zimbabwe.

## Discussion

Effective interventions to drive key outcomes are sorely needed in global development and many other sectors. In this paper, we aim to help programs arrive at an effective portfolio of interventions in two ways.

First, to effectively design interventions that change target behaviors, we introduce a novel and practical framework of behavior, CUBES, to help programs categorize and understand the barriers and enablers, influencers and stages of behavior change ( Figure 1 and Table 2). CUBES synthesizes widely validated evidence across psychology, behavioral economics, market research, and sociology ^7, 8, 11– 14, 16, 19, 20, 22, 35, 41, 48, 51, 56, 76^, and presents its building blocks with a view to actionability by programs. CUBES provides a checklist for programs to systematically assess what is already known about drivers of a target behavior, where novel research is most needed in order to design actionable levers of change, and, after closing evidence gaps, where interventions could focus.

Second, not every type of driver is best measured in the same way. We therefore curate a set of descriptive, experimental, and simulation approaches across sectors, and advocate for a method mix tailored to the gaps in knowledge in a given program ( Figure 2 and Table 2). Some approaches, such as different types of observation and self-report, are already well-established in global development, but using CUBES to structure the components of insight generation ensures that programs can design tools in a systematic way, ultimately saving time and money. For example, quantitative surveys would benefit from the selective incorporation of validated scales to measure specific drivers, or from ways to encourage participants to respond to sensitive topics with greater fidelity. Other methods are well-used in other sectors such as market research, experimental psychology, and decision sciences, and programs could benefit from them for specific purposes. For instance, discrete choice experiments and decision games provide an experimental way to systematically vary and identify key enablers and barriers before testing interventions in the field. Implicit attitude test can be considered as a complementary method to self-report when strong biases are presumed to be at play, and simulations modeling complex systems provide programs with a way to estimate the importance and interaction of multiple drivers, as well as test ‘what-if’ scenarios. We demonstrate the process of choosing methods for specific purposes in a case study on voluntary medical male circumcision uptake ( Figure 3). Of course, data collection in any program will also be influenced by considerations about cost, time, and skill resourcing. There is no hard-and-fast rule of how each method ranks on those three parameters, as much depends on existing organizational and program infrastructure. Nevertheless, we urge programs to estimate these parameters before choosing a methodological path, and to also consider trade-offs in investing upfront versus potential time and cost savings in the intervention phase.

Once data has been collected, the CUBES framework can again be used to structure findings and highlight the specific content and potential targets of an intervention. Creating interventions to fit the varied barriers to behavior is a challenge as well as an opportunity for global health. For example, higher levels of conscientiousness have consistently been associated with higher adherence to medication ^107^ or the contraceptive pill ^108^. Rather than attempting to influence conscientiousness, an intervention might consist of identifying those with lower conscientiousness and targeting increased levels of support to this sub-population. In other situations, drivers may be affected directly. For example, using Facebook to alert college students that peer social norms around drinking were lower than they thought changed drinking behavior ^109^. As an example of targeting a belief, enhancing self-efficacy through encouragement on progress, attribution of progress to participants’ own abilities, observation of others carrying out the target behavior, and other strategies significantly increased physical activity in older adults ^110^. These examples are by no means indicative of success in other contexts and behaviors, and interventions all need to be piloted. However, using a framework of behavior allows for the identification of possibilities that may otherwise remain hidden, and conversely narrow the options for choosing suitable intervention types.

Previously, Michie *et al.* ^111^ provided a comprehensive overview of 93 behavior change techniques, from social comparison to incentives, feedback on behavior, prompts, and goal setting ^111^, as well as a more high-level categorization of nine types intervention from education to training, incentivization, and environmental restructuring ^22^. While identifying mechanisms of actions for interventions remains a work in progress ^112^, these categories can serve as decision aids and an overview of options to programs. The Fogg Model of behavior, as well as the MINDSPACE checklist, also provide a useful classification of what characterizes effective prompts that can increase motivation and ability ^20, 21^.

In many contexts, a single type of intervention may not be enough. We previously showed that psycho-behavioral segmentation can be a powerful method for finely targeting interventions beyond a one-size-fits-all approach ^5, 6, 113^. In the voluntary medical male circumcision program, we used quantitative survey data to segment men on what drove them toward or away from the procedure, and could therefore tailor interventions specifically to each segment ^5^. However, sound and actionable segmentation can only be performed on large-scale quantitative datasets, which is a consideration for the method mix chosen.

After designing interventions to match key barriers found, whether at the population or segment level, we also recommend optimizing and ‘funneling’ interventions from a large pool of potential options down to a narrow set that can be thoroughly evaluated in the field while maximizing the likelihood of success ( Figure 2). Discrete choice experiments can help programs choose between types of interventions and their components at the design stage. Even at the field test stage, factorial designs could test sets of interventions more efficiently, such as different magnitudes of monetary incentives, a distinct messaging component in each, and a different channel through which they are deployed. Recently, this approach has been refined in the Multiphase Optimization Strategy (MOST) for determining the best set of intervention components ^114^. ‘ *In vitro*’ experiments have inherent limitations, as they are not fully replicative of real-world contexts and behaviors that participants are asked to engage in. However, experiments can link a large set of features to an actual behavioral outcome in a controlled way. It is plausible, but yet to be tested systematically, that the closer the ‘ *in vitro*’ behavior to its real-world counterpart, the more informative such experiments might be. For example, being asked to pick among different physical products (such as contraceptive packages) with different features is a task not far removed from reaching for an actual product in a pharmacy. Asking community health workers to pick among job options with varying attributes such as salary, workload, and career progression ^115^ might not be too different from workers weighing those considerations when looking at a job ad. An experiment asking nurses to evaluate and react to a hypothetical emergency, however, might be much more distant from how the choice behavior would play out in a real-life context.

CUBES and the methods toolkit proposed here have several limitations. Frameworks of behavior in general have an ‘evaluation problem’, as it is not feasible to directly compare the insights generated *de novo* using many different behavioral frameworks with very different components. The true test of time will lie in whether programs judge CUBES to be useful in increasing intervention fit, as well as effectiveness at reaching target outcomes, as we have found in our own programs. So far, we have used this ‘utility test’ in our own work, and to give speedy feedback to other organization, in two ways: first, we have used the framework to evaluate planned data collection for comprehensiveness. Second, the framework has been used for dimensionality reduction of expansive datasets (such as household surveys), to enable more clarity in analysis. Future testing should also include critical review of whether intervention options expanded when CUBES was used, and ultimately the change in impact that systematic intervention design yields. A second limitation is that it is unlikely that any one program will be able to draw on expertise for all method options equally, and many techniques require specialized skills to design, field, and analyze. This limitation can be somewhat mitigated by bringing in expert resources, although programs may also face difficulties gathering that network of expertise.

CUBES and permutations of its methods toolkit have now been used in several large-scale programs, from investigating healthcare provider behavior and household behaviors along the maternal and neonatal healthcare pathway in Uttar Pradesh, India, to understanding and influencing tuberculosis care-seeking in South India (unpublished data) and voluntary medical male circumcision in Africa ^5^. We hope that linking the categorization and measurement of enablers and barriers to behavior will enable many more programs to design efficient and effective interventions that get results, and in turn iteratively refine the approaches introduced here.

## Data availability

All data underlying the results are available as part of the article and no additional source data are required.

## Footnotes

^i^United Nations. About the sustainable development goals. Available from: https://www.un.org/sustainabledevelopment/sustainable-development-goals/.

^iii^ Project Implicit Research Group. Project Implicit 2011. Available from: https://implicit.harvard.edu/implicit/ethics.html.
