# Supplementary material for: CUBES: A practical toolkit to measure enablers and barriers to behavior for effective intervention design
Source: Gates Open Res. 2020 Jan 6;3:886. Originally published 2019 Mar 18. [Version 2] doi: 10.12688/gatesopenres.12923.2 (PMC6601426; doi:10.12688/gatesopenres.12923.2)
Supplement: Peer review history

# Peer review: Reviewer response for version 1

**DOI:** 10.21956/gatesopenres.14025.r27004

**Published:** 2019-04-29

**Authors:** Neela A. Saldanha

**Affiliations:** 1 Centre for Social and Behaviour Change (CSBC), Ashoka University, Sonipat, Haryana, India

This is an important and useful article and provides a practical guide to create effective behavior change programs.

 The rationale for this toolkit is clearly stated - behavior change interventions often do not work or are not sustainable. We need to move from a "single-intervention" silver bullet approach to a more strategic program approach comprising multiple interventions. But in the face of audience and context complexities, multiple research tools and disciplines, where do we begin? This article provides that answer, both in the form of a practical behavior change framework and appropriate research methods to address each part of the framework.

 The behavior change framework the authors outline is extremely useful, as practitioners struggle to cobble together multiple frameworks, none of which comprehensively addresses all barriers. In particular, I appreciated the authors explicitly calling out **contextual barriers & enablers** in the model. Most behavior change frameworks do not reference these factors clearly and yet we know from a body of work on behavioral science that such constraints can be a major driver of behavior and especially of the intent-action gap. Having both perceptual and contextual barriers and enablers in the model makes it a complete framework.

 Second, in making the distinction between **habits** or repeated behaviors and one-time behaviors, the authors make the model more clear and effective. Given that habit formation has rarely been a focus in much of development work makes it even more important to explicitly consider whether the behavior we are looking to solve for is a one-time behavior or forming of a habit.

 Third, the curated list of research **techniques** the authors outline is of real value to practitioners who need more of these kinds of lists to enable them to expertly navigate the research world. I know I will certainly turn to this list when designing research.

 Finally, I must commend the authors for strongly calling for more "in-vitro" **experimentation**. Right now, the only evidence-driven work is through multi-year RCTs that are expensive and not flexible and are only in place after resources have already been expended on designing and piloting the program. Having a way to rapidly test elements of the program at the design stage can ensure we are not wasting valuable resources up-front and can course correct early enough.

 As a practitioner, I have a few suggestions to increase the usefulness and likelihood of uptake of the framework by practitioners: 

  

1. **Make it easy: **I would urge the authors to simplify the framework and drive to cognitive ease so that practitioners can easily adopt it. For example, the authors may weigh the benefits of including the "Stages of Change" part of the model against the complexity it introduces for implementation. This could certainly be a more in-depth version of the basic model but in the first instance, defining the behavior well and then understanding perceptual and contextual barriers and enablers to that behavior would go a long way to designing effective programs.
  It would also be helpful to give practitioners some rules of thumb that are actionable. For example, I would recast the narrative of the framework in the form of three simple rules:
  1) Understand determinants of behavior: barriers and enablers, both perceptual and contextual. 
  2) Design idea-channel interventions that address barriers and leverage enablers. 
  3) Design to all levels of change – individual, family, society.
2. **Consider adding the construct of goals & identity to the perceptual barriers and enablers:** All human behavior is goal driven. Goals drive attention and value and are therefore critical to motivation and establishing intent. At the same time, current research on the field prioritizes knowledge and attitudes rather than goals. In doing so, we often conclude research with little idea of this important determinant of motivation - people's goals and how the intended behaviors can help them achieve their goals. The authors mention the construct of goals several times in the article but should consider including this construct in the framework. Similarly a focus on identity, particularly critical in habit formation would add to the richness of the perceptual barriers and enablers. 
3. **Clarify the role of social norms: **It was not intuitively clear to me why social norms which are strongly rooted in psychology are part of contextual factors that has other non-psychological components like laws & infrastructure. 
4. **Create more specifics for each construct:** The overall article does a good job of clearly describing each construct. It would be helpful as a job aid to present a specific checklist for each construct. For example, the different types of beliefs one could uncover in research. Practitioners often don’t know all the theoretical underpinnings of a construct and may use their own interpretation. This would result over time in the constructs being interpreted differently and therefore reduce the scope for cross-learning across programs. 
5. **Create more detail and specificity on the design process**: The article has a lot of emphasis on the research process to generate insight. There is a much shorter section on effective behavioral design, though this is often the stage when practitioners fail to convert rich insight into effective ideas. Similar to the detailed manner in which the research techniques have been outlined, it would be useful to outline the design process with appropriate checklists. For example how to prioritize ideas, how to effectively prototype prioritized ideas, ways to rapidly test ideas ranging from experiments to rapid qualitative feedback loops and other such steps in the process. Within this design process, there needs to be a clear understanding of the process to design mass media communication as often mass media & non mass media interventions are created separately rather than as part of one integrated program. 

Overall, this is a very practical and clear article. I hope the suggestions I have laid out are useful to the authors and that this work can lead to more such best practices which can help interventionists design effective programs.

I confirm that I have read this submission and believe that I have an appropriate level of expertise to confirm that it is of an acceptable scientific standard.

# Response

**Published:** 2019-12-18

**Authors:** Sema Sgaier

We thank the reviewer for the positive evaluation of and detailed comments on our work. We appreciate the comments’ focus on increasing the likelihood of the framework’s uptake by practitioners, and have adapted the article in the following ways:

**1. Make it easy: I would urge the authors to simplify the framework and drive to cognitive ease so that practitioners can easily adopt it. For example, the authors may weigh the benefits of including the "Stages of Change" part of the model against the complexity it introduces for implementation. This could certainly be a more in-depth version of the basic model but in the first instance, defining the behavior well and then understanding perceptual and contextual barriers and enablers to that behavior would go a long way to designing effective programs.**

**It would also be helpful to give practitioners some rules of thumb that are actionable. For example, I would recast the narrative of the framework in the form of three simple rules:**

**1) Understand determinants of behavior: barriers and enablers, both perceptual and contextual.**

**2) Design idea-channel interventions that address barriers and leverage enablers.**

**3) Design to all levels of change – individual, family, society.**

The balance between simplicity and depth is indeed challenging, and we have faced this both internally and when working with practitioners from other organizations. Ultimately, we believe that in this manuscript, there is value in retaining complexity of the drivers at the current level. For example, the present Stages of Change have already been reduced from more nuanced stages in the Transtheoretical Model, and we believe that understanding behavior as a journey from awareness/skills to intention to action and beyond is key to creating successful interventions that highlight that awareness is not enough. However, where we fully agree with this comment is that we need to create better tools to help practitioners engage with the toolkit. Therefore, we have now initiated building a website where we will provide more in-depth and animated walk-throughs of the toolkit, in addition to further case studies.

We also appreciate the actionable three rules of thumb re-framing the narrative of CUBES, and have incorporated them into the Introduction in a slightly modified form:

*Ultimately, we encourage practitioners use the toolkit to:*

1. *Understand determinants of behavior: barriers and enablers, both perceptual and contextual. *
2. *Design idea-channel interventions that address barriers and leverage enablers. *
3. *Design to all levels of change – individual, family, society, and systems.*

**2. Consider adding the construct of goals & identity to the perceptual barriers and enablers: All human behavior is goal driven. Goals drive attention and value and are therefore critical to motivation and establishing intent. At the same time, current research on the field prioritizes knowledge and attitudes rather than goals. In doing so, we often conclude research with little idea of this important determinant of motivation - people's goals and how the intended behaviors can help them achieve their goals. The authors mention the construct of goals several times in the article but should consider including this construct in the framework. Similarly a focus on identity, particularly critical in habit formation would add to the richness of the perceptual barriers and enablers.** 

These are highly important points. We agree with the importance of seeing behavior as goal-directed, but have not included it as a separate construct, as it folds into the construct of ‘intention’. While goals and intentions are not equivalent concepts, we have now clarified in the Results section and the legend of Figure 1 that intention can mean a plan of action *towards* a specific goal (Ajzen I, Madden TJ. Prediction of goal-directed behavior: Attitudes, intentions, and perceived behavioral control. Journal of Experimental Social Psychology. 1986;22: 453–474):

*The CUBES framework therefore divides the behavioral change process more simply into three stages of knowledge (ending with the necessary awareness or skills to engage in a behavior), intention (which can also be understood as a plan of action towards a specific goal), and action.*

For intervention design, research on specifics of goal-setting has provided fascinating insights: for instance, specific goals are better than vague encouragements to ‘do one’s best’, goals should be challenging yet achievable, and time-proximal goals are more effective than goals far in the future (Locke EA, Latham GP. Building a practically useful theory of goal setting and task motivation: A 35-year odyssey. American Psychologist. 2002;57: 705–717). We therefore agree that incorporating the concept of goals into intentions is very useful, although, as we mention below, in this article we cannot focus on the mechanics of intervention design.

We had originally not incorporated the concept of ‘identity’ (such as professional or social identity) into the CUBES framework for two reasons: first, identity can refer to a set of behaviors, personal qualities, or internal beliefs (Cane J, O’Connor D, Michie S. Validation of the theoretical domains framework for use in behaviour change and implementation research. Implementation Science. 2012;7). For intervention design, identity is often thought of more simply as core beliefs about oneself. Second, it is exciting that there are now several studies showing the predictive value of an identity concept on behavior (for example: van der Werff E, Steg L, Keizer K. The value of environmental self-identity: The relationship between biospheric values, environmental self-identity and environmental preferences, intentions and behaviour. Journal of Environmental Psychology. 2013;34: 55–63; Nigbur D, Lyons E, Uzzell D. Attitudes, norms, identity and environmental behaviour: Using an expanded theory of planned behaviour to predict participation in a kerbside recycling programme. British Journal of Social Psychology. 2010;49: 259–284). However, the evidence base on the concept’s predictiveness on behavior is still inconsistent, and too limited in comparison to other types of beliefs to recommend its inclusion into the CUBES framework at this point. We do, however, now mention the emerging evidence in the manuscript (Results):

*Beliefs around (professional or social) self-identity may also be predictive of behavior. For example, environmental self-identity, or seeing oneself as ‘a person who acts environmentally-friendly’, is related to several environmental behaviors (van der Werff E, Steg L, Keizer K. The value of environmental self-identity: The relationship between biospheric values, environmental self-identity and environmental preferences, intentions and behaviour. Journal of Environmental Psychology. 2013;34: 55–63). However, empirical research on identity and behavior is still emerging.*

**3. Clarify the role of social norms: It was not intuitively clear to me why social norms which are strongly rooted in psychology are part of contextual factors that has other non-psychological components like laws & infrastructure.**

We thank the reviewer for bringing our attention to this important point. We have now further clarified our rationale in the ‘Results – Contextual drivers of behavior’ section:

*Social norms are a construct that can only exist on the level outside the individual: through collective behavior and ‘shared knowledge’, norms describe a set of practices of what other people do (descriptive norms), or prescribe what people should do (prescriptive norms). Both of these may influence attitudes and behavior (Smith JR, Terry DJ, Manstead ASR, Louis WR, Kotterman D, Wolfs J. The attitude–behavior relationship in consumer conduct: the role of norms, past behavior, and self-identity. The Journal of Social Psychology. 2008;148: 311–334). Unlike individual-level beliefs, norms usually imply some consequence to the individual should they deviate from the norm, such as disapproval (Brauer M, Chaurand N. Descriptive norms, prescriptive norms, and social control: an intercultural comparison of people’s reactions to uncivil behaviors. Eur J Soc Psychol. 2010;40: 490–499).*

**4. Create more specifics for each construct: The overall article does a good job of clearly describing each construct. It would be helpful as a job aid to present a specific checklist for each construct. For example, the different types of beliefs one could uncover in research. Practitioners often don’t know all the theoretical underpinnings of a construct and may use their own interpretation. This would result over time in the constructs being interpreted differently and therefore reduce the scope for cross-learning across programs.** 

We fully agree with the reviewer’s point that constructs should be defined in greater depth, and indeed they have by the theoretical literature on which this toolkit has been built. We see the most value in building out additional guidance in the form of examples most likely to be useful to practitioners, such as questions that have been or could be asked in surveys to capture constructs. This is a longer-term process, and we aim to incorporate this guidance in a more interactive form on the upcoming web platform.

**5. Create more detail and specificity on the design process: The article has a lot of emphasis on the research process to generate insight. There is a much shorter section on effective behavioral design, though this is often the stage when practitioners fail to convert rich insight into effective ideas. Similar to the detailed manner in which the research techniques have been outlined, it would be useful to outline the design process with appropriate checklists. For example how to prioritize ideas, how to effectively prototype prioritized ideas, ways to rapidly test ideas ranging from experiments to rapid qualitative feedback loops and other such steps in the process. Within this design process, there needs to be a clear understanding of the process to design mass media communication as often mass media & non mass media interventions are created separately rather than as part of one integrated program.**

In this article, we have aimed to provide an overview of how to systematize potential drivers of behavior and methodologies to generate insights around them. We agree that the ultimate goal is to enable programs to design interventions that address key barriers and leverage main enablers. However, the focus of this article could not be on the specifics around intervention design, for several reasons. One reason is that reviewing the evidence on the intervention types that work or don’t in different contexts cannot be done justice in a single article. For example, once a specific social norm has been identified as a barrier to a target behavior, would it work best to leverage key influencers to change the norm, create mass media entertainment showing peer role models (as in an intervention in Rwanda around that used a soap opera to model inter-group harmony; Staub E, Pearlman LA. Reducing intergroup prejudice and conflict: A commentary. Journal of Personality and Social Psychology. 2009), or something else entirely? Behavioral intervention design is its own vast and ongoing area of research and trial and error, and here we can only encourage practitioners to do their own research on potentially effective approaches, once they have used the CUBES toolkit to zero in on the barriers to solve for. However, we have now added some examples of how behavioral drivers have been or could be addressed to the Discussion (see response to the first comment by Reviewer 2). Second, for detail on how to conduct rapid prototyping of solutions, we believe that practitioners of human-centered design, and the frameworks by development partners (such as JHU-CCP, PSI, and FHI360) raised in the second point by Reviewer 1, already provide rich guidance. In contrast, we think this manuscript will be most impactful if focused on categorizing and measuring drivers of behavior.

# Peer review: Reviewer response for version 1

**DOI:** 10.21956/gatesopenres.14025.r27007

**Published:** 2019-04-01

**Authors:** Rashida A. Ferrand

**Affiliations:** 1 London School of Hygiene and Tropical Medicine, London, UK; 2 Biomedical Research and Training Institute, Harare, Zimbabwe

This paper describes an approach to comprehensively consider drivers of behaviour change to inform effective intervention design. 

 The authors propose a framework that takes into account both perceptual and contextual factors as well as influencers of behaviour building on available models. This is a refreshing, much needed approach if behavioural interventions are to be effective and lead to impact. I particularly like the emphasis on "repeat and habit" in their framework, in the stages of change. 

 Issues:

- I am not convinced by some of the approaches (and their use) that the authors present for informing interventions in global health and development. While particular factors may be important, how they can be measured and modified needs further thought. For example, the authors mention use of personality tests and measurement of perceptual barriers; incorporating this in intervention design is not that easy particularly in public health interventions. It is important to consider *how* the findings can be used in intervention design. 
- The authors need to consider the limitations of "in vitro" experiments. Behaviour, as the authors point out, is complex with multiple mediators and in vitro experiments may be subject to the same limitations as the approaches used to date, particularly if some factors in the stages of changes are not amenable to change. The issues around effectiveness versus efficacy are still critical in this context.
- The authors present two approaches they have used: 1) in Zimbabwe and Zambia to scale up VMMC and 2) in India on TB care-seeking and maternal and child care. They mention that they have found effectiveness in their own programmes. Disappointingly, there is no evidence presented of the effectiveness of the approach used - the approach in Zimbabwe was published in 2016 and the authors state that it is being piloted nationally in Zimbabwe in 2019. Is there preliminary data on uptake etc.? Available data from DHS etc. in fact suggest that that VMMC rates have fallen in this region. Similarly, the authors present no evidence of the effectiveness of the framework and methods they present. Can the authors demonstrate evidence of the effectiveness of these approaches?
- Limitations about the feasibility of using these approaches and their scientific validity and the feasibility of the findings from these approaches to inform and "modify" the barriers and enablers needs mention and discussion. Ultimately these are the factors that will determine whether the CUBES model is useful. 

I confirm that I have read this submission and believe that I have an appropriate level of expertise to confirm that it is of an acceptable scientific standard, however I have significant reservations, as outlined above.

# Response

**Published:** 2019-12-18

**Authors:** Sema Sgaier

We thank the reviewer for the detailed and useful comments on our article, and for highlighting that this approach to approaching behavioral interventions is much-needed. Taking the different points of feedback into account, we have amended the article in the following ways:

**1. I am not convinced by some of the approaches (and their use) that the authors present for informing interventions in global health and development. While particular factors may be important, how they can be measured and modified needs further thought. For example, the authors mention use of personality tests and measurement of perceptual barriers; incorporating this in intervention design is not that easy particularly in public health interventions. It is important to consider *how* the findings can be used in intervention design. ** 

We fully agree that measuring and creating interventions around a more comprehensive set of behavioral drivers is not trivial. We think there are two parts to this comment. One is on the research methods themselves. In this article, we have already given nuanced intentions around these approaches: we do not aim to claim that all methods are equally useful, informative, and validated for all global health and development use cases, or even overall. However, we outline a set of options and choices, all of which have been developed, used, and validated in other sectors.

The second part is on then developing interventions that fit the broader spectrum of barriers found, where we agree addressing some barriers may be harder than others, but perhaps the bigger question should be about how *useful* it is to address such barriers. While this manuscript does not address intervention design itself, we mention one approach to categorizing types of interventions by behavioral drivers in the Discussion (Michie S, Richardson M, Johnston M, Abraham C, Francis J, Hardeman W, *et al*. The behavior change technique taxonomy (v1) of 93 hierarchically clustered techniques: building an international consensus for the reporting of behavior change interventions. Annals of Behavioral Medicine. 2013;46(1):81-95.). In the Discussion, we have now added more detail around what interventions addressing perceptual drivers could look like:

*Creating interventions to fit the varied barriers to behavior is a challenge as well as an opportunity for* *global health. For example, higher levels of conscientiousness have consistently been associated with higher adherence to medication (Molloy GJ, O’Carroll RE, Ferguson E. Conscientiousness and medication adherence: a meta-analysis.* *Annals of Behavioral Medicine. 2014;47: 92–101) or the contraceptive pill (Leahy D, Treacy K, Molloy GJ. Conscientiousness and adherence to the oral contraceptive pill: a prospective study. Psychology & Health. 2015;30: 1346–1360). Rather than attempting to influence conscientiousness, an intervention might consist of identifying those with lower conscientiousness and targeting increased levels of support to this sub-population. In other situations, drivers may be affected directly. For example, using Facebook to alert college students that peer social norms around drinking were lower than they thought changed drinking behavior (Ridout B, Campbell A. Using Facebook to deliver a social norm intervention to reduce problem drinking at university: social norm intervention using Facebook. Drug Alcohol Rev. 2014;33). As an example of targeting a belief, enhancing self-efficacy through encouragement on progress, attribution of progress to participants’ own abilities, observation of others carrying out the target behavior, and other strategies significantly increased physical activity in older adults (Allison MJ, Keller C. Self-efficacy intervention effect on physical activity in older adults. West J Nurs Res. 2004;26: 31–46). These examples are by no means indicative of success in other contexts and behaviors, and interventions all need to be piloted. However, using a framework of behavior allows for the identification of possibilities that may otherwise remain hidden, and conversely narrow the options for choosing suitable intervention types.*

**2. The authors need to consider the limitations of "in vitro" experiments. Behaviour, as the authors point out, is complex with multiple mediators and in vitro experiments may be subject to the same limitations as the approaches used to date, particularly if some factors in the stages of changes are not amenable to change. The issues around effectiveness versus efficacy are still critical in this context.**

We agree with the reviewer that ‘in vitro’ experiments have inherent limitations, as they are not fully replicative of real-world behaviors in their rich context. We strongly argue that this limitation is different from those of approaches used to date. ‘In vitro’ experimental approaches provide additional data on which interventions to scale up, as experiments uniquely link controlled inputs to actual behavioral outcomes. Furthermore, a larger set of drivers can be incorporated and tested more rapidly and on a smaller sample than in a field RCT. However, the reviewers raise an excellent point on the validity of ‘in vitro’ approaches regarding behavior change. We have now added more nuanced, but speculative, guidance on the kinds of behaviors that might especially benefit from ‘in vitro’ testing to the Discussion:

*‘In vitro’ experiments have inherent limitations, as they are not fully replicative of real-world contexts and behaviors that participants are asked to engage in. However, experiments can link a large set of features to an actual behavioral outcome in a controlled way. It is plausible, but yet to be tested systematically, that the closer the ‘in vitro’ behavior to its real-world counterpart, the more informative such experiments might be. For example, being asked to pick among different physical products (such as contraceptive packages) with different features is a task not far removed from reaching for an actual product in a pharmacy. Asking community health workers to pick among job options with varying attributes such as salary, workload, and career progression (Abdel-All M, Angell B, Jan S, Howell M, Howard K, Abimbola S, et al. What do community health workers want? Findings of a discrete choice experiment among Accredited Social Health Activists (ASHAs) in India. BMJ Global Health. 2019;4) might not be too different from workers weighing those considerations when looking at a job ad. An experiment asking nurses to evaluate and react to a hypothetical emergency, however, might be much more distant from how the choice behavior would play out in a real-life context.*

**

**3. The authors present two approaches they have used: 1) in Zimbabwe and Zambia to scale up VMMC and 2) in India on TB care-seeking and maternal and child care. They mention that they have found effectiveness in their own programmes. Disappointingly, there is no evidence presented of the effectiveness of the approach used - the approach in Zimbabwe was published in 2016 and the authors state that it is being piloted nationally in Zimbabwe in 2019. Is there preliminary data on uptake etc.? Available data from DHS etc. in fact suggest that that VMMC rates have fallen in this region. Similarly, the authors present no evidence of the effectiveness of the framework and methods they present. Can the authors demonstrate evidence of the effectiveness of these approaches?**

We appreciate the reviewer’s point and fully agree that in this manuscript, the effects, benefits and drawbacks of using the framework have not been discussed in enough detail. This is for two reasons: in the VMMC program, we (the authors) are not involved in the national scale-up and associated data analysis and publication. In our own programs, we and our partners are currently in the process of publishing stand-alone papers where we discuss the approach taken in detail. In particular, in these publications we plan to show how using a structured approach to behavioral drivers enabled data collection and analytic approaches that resulted in finding a) nuanced and actionable predictors of behavioral outcomes, b) distinct segments within the population, and c) a much more specific and segment-tailored space for potential interventions. To caveat this, impact evaluations, i.e. designing these interventions, implementing them, and measuring their effect on behavior or even health outcomes, is a much longer-term endeavor. We are fully transparent that it will take many years and multiple stakeholders to get there.

**4. Limitations about the feasibility of using these approaches and their scientific validity and the feasibility of the findings from these approaches to inform and "modify" the barriers and enablers needs mention and discussion. Ultimately these are the factors that will determine whether the CUBES model is useful.**

We believe we have outlined, in the brief space available in a single manuscript, the scientific validity (in terms of predicting behavior) behind the behavioral drivers incorporated in the CUBES framework. Detailed nuances, such as sub-categories of drivers, or their usefulness to predict some behaviors more than others, can be found in the vast underlying literature, of which this manuscript can only provide a concise synthesis. It would also go beyond the scope of this manuscript provide an evaluation of each research method in terms of either feasibility or validity. Both concepts are nuanced. For example, feasibility may encompass dimensions such as time, cost, and implementation, design, or analytic skills required. All of these can vary by context, requirements according to research questions and representativeness required, and existing organizational capability. Likewise, the devil is in the detail when it comes to scientific validity of a research method: it would be impossible to say, for example, that quantitative surveys are either scientifically valid or invalid (or anything in between). Instead, the soundness of an item included in a survey will depend on tests of item comprehension, reliability, and validity, and can depend on details such as the polarity of a rating scale used. We therefore encourage readers to take this toolkit as a starting point to judge whether a method might be suitable for their needs, and then use the body of existing literature to go in depth.

# Peer review: Reviewer response for version 1

**DOI:** 10.21956/gatesopenres.14025.r27005

**Published:** 2019-03-28

**Authors:** Hope Hempstone, Angela Brasington

**Affiliations:** 1 United States Agency for International Development (USAID), Washington, DC, USA; 2 United States Agency for International Development (USAID), Washington, DC, USA

In general, the article is clear and detailed, making a compelling case for further exploration of the CUBES model. The inclusion of concepts from behavioral economics, in particular, represents a clear departure from other models in common use in global health, and will support ongoing efforts to ensure that behavior change programming reflects current and emerging thinking in behavioral science. Furthermore, the authors’ effort to not only describe drivers of behavior, but to present those research methods most appropriate for measuring different drivers, will help render this model actionable and support research-to-practice linkages in behavior change programming. This having been said, the article’s rich content - a new theoretical model and a set of under-used tools for measuring behavioral drivers - is worthy of two articles. Each content area has the potential to contribute significantly to behavior change programming; combining them renders them less digestible. We hope that the authors will consider developing a more detailed publication on one or both components moving forward.

 We note a number of areas for potential improvement below:

1. While the authors acknowledge the small scale on which this model has been tested in their discussion of limitations, doing so earlier in the article - and discussing how best the model might be further applied within Surgo’s work or socialized externally - would help highlight potential for continuing to test and refine CUBES.
2. The authors do themselves a disservice by overstating the novelty of the CUBES model. Many development partners follow a structured process to diagnose and address primary drivers of a behavior of interest, be they individual, social, or structural. A number of development partners, including organizations like JHU-CCP, PSI, and FHI360, employ models of behavior change similar to CUBES, which synthesize more narrowly focused theories and models. It would be helpful if the authors acknowledged the existence of these models, and articulated how CUBES builds upon and departs from them.
3. Decisions around formative research are driven in many settings by not only theory and methodology, but also by practical concerns regarding cost, speed of implementation, and availability of trained researchers. Acknowledging this reality, and discussing how the proposed approaches might impact these factors, would be helpful to external readers as they consider how best to apply CUBES in the context of their programs.
4. The discussion of social influence could be strengthened (clarified) in a number of key ways. First, the language used implies that influencers are the source of behavioral barriers and enablers (“...these barriers and enablers may be transmitted to the individual through influencers either directly or indirectly or through media channels.”). With the exception of behaviors that are strongly (primarily?) influenced by social norms and real or perceived social support, this would seem to be an inaccurate characterization of social influence; would it be clearer and more accurate to note that barriers and enablers are reinforced or contradicted by influencers? The authors also seem to imply that social influence is typically explicit and intentional (as in the context of women’s groups, for example), when in fact this is often not the case. Acknowledging the widely varied forms that social influence can take would be helpful to implementers in understanding it as a driver of behavior and considering how best to leverage influencers in interventions.
5. The authors note that one-time and periodic behaviors differ from habitual behaviors, and that different types of interventions may be required to change behaviors depending on their attributes. Attribute-based grouping of behaviors is an area of growing interest among researchers, donors, and behavior change implementers; the authors might consider acknowledging this fact as it has bearing on continued application and refinement of models like CUBES, particularly in the context of multi-health element behavior change programming.
6. The article could be strengthened by more focused attention to the role of emotion in behavior. This driver, in particular, seems to be poorly understood by implementers and researchers; additional guidance on how best to measure emotion would be of tremendous practical utility.
7. The authors and their organization have been vocal advocates for improved audience segmentation in global health programming, and in practice identification of priority behavioral determinants goes hand-in-hand with segmentation of audiences (through doer/non-doer analysis and similar design activities). It would be very helpful if the authors could provide additional detail about the relationship between segmentation and the identification and prioritization of determinants.
8. We beg to differ with the assertion made on p. 13 of the article that randomized control trials predominate in development programming. In our experience, RCTs are employed in only a small minority of programs, particularly in the case of behavior change programming. Recognizing this (and other) practical realities, and addressing them directly, will lend credence to the authors’ position.

We confirm that we have read this submission and believe that we have an appropriate level of expertise to confirm that it is of an acceptable scientific standard.

# Response

**Published:** 2019-12-18

**Authors:** Sema Sgaier

We thank the reviewers for taking the time to thoroughly evaluate this article, and for their positive comments. In particular, we are glad the reviewers consider the framework actionable. Taking their feedback into account, we have amended the article in the following ways:

**1. While the authors acknowledge the small scale on which this model has been tested in their discussion of limitations, doing so earlier in the article - and discussing how best the model might be further applied within Surgo’s work or socialized externally - would help highlight potential for continuing to test and refine CUBES.**

We fully agree with the reviewers that continued refinement and testing are crucial to any framework’s development. Several levels of testing could conceivably be done, adding different types of value. First, the framework can be assessed on its data collection utility: does it fulfil the promise of speedier data collection development through offering a skeleton of constructs to collect? Through our internal studies (currently in the process of being published), we have found the framework to be helpful in that regard, and have also used it to give rapid feedback on other organizations’ research plans.

A second consideration is whether data generated using the framework generates richer insights that increase the intervention options available to programs. In fact, this point was a premise of creating the framework in the first place, as it is plausible that if fewer categories are measured, fewer types of interventions can be matched to them. However, the success of matching distinct intervention types to distinct enablers and barriers will need to be evaluated through a large set of use cases from many organizations.

Third, there is the question of whether the constructs used in CUBES, taken together, are more predictive of behaviors than those defined in other frameworks. Testing this question is a life-time challenge for researchers and has not been the purview of our work, but we aimed to maximize the likelihood by incorporating only constructs based on a substantial evidence base.

Rather than earlier in the article, we have expanded on these considerations in the Discussion, where we had already touched on them:

*The true test of time will lie in whether programs judge CUBES to be useful in increasing intervention fit, as well as effectiveness at reaching target outcomes, as we have found in our own programs. So far, we have used this ‘utility test’ in our own work, and to give speedy feedback to other organization, in two ways: first, we have used the framework to evaluate planned data collection for comprehensiveness. Second, the framework has been used for dimensionality reduction of expansive datasets (such as household surveys), to enable more clarity in analysis. Future testing should also include critical review of whether intervention options expanded when CUBES was used, and ultimately the change in impact that systematic intervention design yields.*

**2. The authors do themselves a disservice by overstating the novelty of the CUBES model. Many development partners follow a structured process to diagnose and address primary drivers of a behavior of interest, be they individual, social, or structural. A number of development partners, including organizations like JHU-CCP, PSI, and FHI360, employ models of behavior change similar to CUBES, which synthesize more narrowly focused theories and models. It would be helpful if the authors acknowledged the existence of these models, and articulated how CUBES builds upon and departs from them.**

We agree with this important point, and have included the JHU-CCP, PSI, and FHI360 models in the Introduction’s overview of behavioral models (however, not in Table 1, as their behavioral driver components are derived from other models):

*In global development, several organizations such as PSI, Johns Hopkins’ Center for Communication Programs, and FHI360 have created behavioral models incorporating various subsets of drivers. Being application-focused, they usually place great focus on incorporating guidelines on implementation design and monitoring, communication, and advocacy, or on providing a rich compendium of intervention options.*

As the reviewers note, these models also incorporate evidence-based drivers of behavior, but are less focused on helping programs systematizing and measuring them. No less importantly, they tend to be more focused than CUBES on either the process of creating change by incorporating guidelines on implementation design and monitoring, communication, and advocacy, or on providing a rich compendium of intervention options. For example, FHI360’s ‘C-Change’ Social and Behavior Change Communication Framework builds mostly on the socio-ecological model and lays out the process of changes management, incorporating some perceptual drivers, but not contextual ones. The Johns Hopkins Center for Communication Programs’ (JHU-CCP) Pathways framework, in contrast, starts from laying out different intervention types and what behavioral shifts on individual and community levels can be expected from them. JHU-CCP have also built many successful interventions based on nudges that aim to shift implicit biases, such as commitment devices. PSI’s ‘Bubbles’ Behavior Change Framework is closely related to the COM-B framework, and includes many important drivers, but does not focus on context or influencers and channels.

**3. Decisions around formative research are driven in many settings by not only theory and methodology, but also by practical concerns regarding cost, speed of implementation, and availability of trained researchers. Acknowledging this reality, and discussing how the proposed approaches might impact these factors, would be helpful to external readers as they consider how best to apply CUBES in the context of their programs.**

We also agree with the reviewers that decisions about research are impacted by cost, speed, and capability considerations. For this article, we deliberately decided not to go into detail on these points, as the variability in program context, organizational structure, and method chosen all greatly impact these three parameters without many hard-and-fast rules. For example, there cannot be a blanket assessment of whether conducting a quantitative survey or qualitative ethnography are more costly, lengthy, or require additional skills, as it depends on where each program is at a given point in time. Instead, we have added the general point to the Discussion that the three parameters should be considered in tandem at the start of a research process, and that initial resource investment should be compared to later payoffs:

*Of course, data collection in any program will also be influenced by considerations about cost, time, and skill resourcing. There is no hard-and-fast rule of how each method ranks on those three parameters, as much depends on existing organizational and program infrastructure. Nevertheless, we urge programs to estimate these parameters before choosing a methodological path, and to also consider trade-offs in investing upfront versus potential time and cost savings in the intervention phase.*

**4. The discussion of social influence could be strengthened (clarified) in a number of key ways. First, the language used implies that influencers are the source of behavioral barriers and enablers (“...these barriers and enablers may be transmitted to the individual through influencers either directly or indirectly or through media channels.”). With the exception of behaviors that are strongly (primarily?) influenced by social norms and real or perceived social support, this would seem to be an inaccurate characterization of social influence; would it be clearer and more accurate to note that barriers and enablers are reinforced or contradicted by influencers? The authors also seem to imply that social influence is typically explicit and intentional (as in the context of women’s groups, for example), when in fact this is often not the case. Acknowledging the widely varied forms that social influence can take would be helpful to implementers in understanding it as a driver of behavior and considering how best to leverage influencers in interventions**.

The reviewers make an excellent point regarding the clarification of the language around social influence. We agree that influencers can reinforce or contradict enablers and barriers. We think it is not mutually exclusive to say they may also be the source of enablers and barriers: for example, influencers may formulate and transmit certain beliefs. We also agree that social influence is varied and may be explicit and intentional as well as, likely more commonly, implicit. We have now highlighted that variability in the Results section:

*Third, these barriers and enablers may be transmitted to the individual, reinforced, or weakened through influencers (such as friends, family, or community members), either directly or through media channels…*

*Influencers can reach individuals either directly or at scale via various media channels, which is important information to determine how to deliver interventions. For example, female self-help groups can serve as a channel for rural women in India to reinforce or change social norms relating to a certain target behavior. For an intervention to work, the content, the type of influencer, and the channel through which they reach individuals must be identified as relevant to the target individuals. Finally, social influence is often not intentional as a self-help group might be, but less explicit influence may not be any less powerful.*

**5. The authors note that one-time and periodic behaviors differ from habitual behaviors, and that different types of interventions may be required to change behaviors depending on their attributes. Attribute-based grouping of behaviors is an area of growing interest among researchers, donors, and behavior change implementers; the authors might consider acknowledging this fact as it has bearing on continued application and refinement of models like CUBES, particularly in the context of multi-health element behavior change programming.**

The reviewers’ point that attribute-based grouping of behaviors (for example, whether they are one-time or habitual) leads to different types of interventions is well-taken. Intervention design would also be influenced by other types of attributes, such as whether the behavior to be changed is one of commission (doing more of something) omission (refraining from doing something). However, considering the focus of this article – structuring and measuring drivers of behavior – we believe that this would merit a much more detailed exploration in its own article, with a greater focus on intervention design itself.

**6. The article could be strengthened by more focused attention to the role of emotion in behavior. This driver, in particular, seems to be poorly understood by implementers and researchers; additional guidance on how best to measure emotion would be of tremendous practical utility.**

We fully agree with the reviewers that emotion is a particularly under-measured driver, and that existing methods do not often do a good job capturing it. While this may partly be because of a lack of commitment to capture data about emotion using standard methods such as observation or surveys, the science of measuring emotion is also still very much in development. This is due to the many distinct components and theories of emotion. For example, emotion has an arousal component, which may be picked up using physiological measures such as eye tracking or heart rate. However, arousal does not tell us about valence, or in simple terms whether the arousal is positive (such as excitement) or negative (such as anxiety). Facial expression coding is another possible avenue of emotion measurement, and several private-sector companies now claim to automate emotion labeling from people’s facial expressions, for example while they watch a target video. However, there remains a vigorous scientific debate on what these feature detectors actually measure. These methods are also difficult to implement in a field context. For the moment, we believe that adding non-verbal scales, such as those described in the Standardized Scales section, sentiment analysis from speech or text data, as well as simply being conscious of including self-reported emotion in questionnaires, provide the clearest return on investment for programs.

**7. The authors and their organization have been vocal advocates for improved audience segmentation in global health programming, and in practice identification of priority behavioral determinants goes hand-in-hand with segmentation of audiences (through doer/non-doer analysis and similar design activities). It would be very helpful if the authors could provide additional detail about the relationship between segmentation and the identification and prioritization of determinants.**

We completely agree with the reviewers that identifying priority behavioral determinants and segmentation often go hand in hand. However, they are not necessarily the same. For example, in some situations it is conceivable that there are barriers to a target behavior that are more or less universal to the population. We usually see segmentation as a next step following from the identification of behavioral drivers within a population: when these data are known on an individual level, segmentation can then reveal sub-groups in the population, and how drivers are differently weighed within them. Behavioral drivers themselves can therefore still be seen as the ‘building blocks’ of segmentation: for example, one segment of community health workers might have high knowledge and a high self-efficacy being supported by her supervisors and villagers alike, but faces infrastructure problems reaching her village constituents; in another segment, the barriers might be reversed. Our recent methods article on segmentation goes into great detail on how to link the framework of behavior and segmentation, and can be accessed here. 

We have added this new reference to the Discussion.

**8. We beg to differ with the assertion made on p. 13 of the article that randomized control trials predominate in development programming. In our experience, RCTs are employed in only a small minority of programs, particularly in the case of behavior change programming. Recognizing this (and other) practical realities, and addressing them directly, will lend credence to the authors’ position.**

While we agree with the reviewers that RCTs do not predominate in practice, we do find that they are seen as the gold standard of evidence, particularly in interventions based on behavioral economics. This may mean programs are missing out on in vitro testing approaches. We have changed the language in the section ‘Results - Choosing the right method at the right time for the right purpose’ to be more nuanced in that regard:

*In global development, many programs tend to focus on descriptive methods for insight generation, followed by field implementation. In the field, randomized controlled trials tend to be seen as the gold standard for assessing the effectiveness of an intervention, even if they are not always employed in practice.*
